# TGF-β signaling promotes tube-structure-forming growth in pancreatic duct adenocarcinoma

**DOI:** 10.1038/s41598-019-47101-y

**Published:** 2019-08-02

**Authors:** Takashi Yamaguchi, Sanae Ikehara, Yoshihiro Akimoto, Hayao Nakanishi, Masahiko Kume, Kazuo Yamamoto, Osamu Ohara, Yuzuru Ikehara

**Affiliations:** 1 Department of Molecular and Tumor Pathology, Graduate School of Medicine, Chiba University, Chiba, 260-8670 Japan; 2 Biotechnology Research Institute for Drug Discovery, National Institute of Advanced Industrial Science and Technology (AIST), Tsukuba, 305-8565 Japan; 3 Department of Anatomy, Kyorin University School of Medicine, Mitaka, 181-8611 Japan; 4 Laboratory of Pathology and Clinical Research, Aichi Cancer Center Aichi Hospital, Okazaki, 444-0011 Japan; 5 Department of Integrated Biosciences, Graduate School of Frontier Sciences, The University of Tokyo, Kashiwa, 277-8562 Chiba, Japan; 6 Department of Applied Genomics, Kazusa DNA Research Institute, Kisarazu, 292-0818 Japan

**Keywords:** Morphogen signalling, Pancreatic cancer, Metastasis, Cell invasion, Epithelial-mesenchymal transition

## Abstract

Tube-forming growth is an essential histological feature of pancreatic duct adenocarcinoma (PDAC) and of the pancreatic duct epithelium; nevertheless, the nature of the signals that start to form the tubular structures remains unknown. Here, we showed the clonal growth of PDAC cell lines in a three-dimensional (3D) culture experiment that modeled the clonal growth of PDAC. At the beginning of this study, we isolated the sphere- and tube-forming clones from established mouse pancreatic cancer cell lines via limiting dilution culture using collagen gel. Compared with cells in spherical structures, the cells in the formed tubes exhibited a lower CK19 expression in 3D culture and in the tumor that grew in the abdominal cavity of nude mice. Conversely, the expression of the transforming growth factor β (TGF-β)-signaling target mRNAs was higher in the formed tube vs the spherical structures, suggesting that TGF-β signaling is more active in the tube-forming process than the sphere-forming process. Treatment of sphere-forming clones with TGF-β1 induced tube-forming growth, upregulated the TGF-β-signaling target mRNAs, and yielded electron microscopic findings of a fading epithelial phenotype. In contrast, the elimination of TGF-β-signaling activation by treatment with inhibitors diminished the tube-forming growth and suppressed the expression of the TGF-β-signaling target mRNAs. Moreover, upregulation of the Fn1, Mmp2, and Snai1 mRNAs, which are hallmarks of tube-forming growth in PDAC, was demonstrated in a mouse model of carcinogenesis showing rapid progression because of the aggressive invasion of tube-forming cancer. Our study suggests that the tube-forming growth of PDAC relies on the activation of TGF-β signaling and highlights the importance of the formation of tube structures.

## Introduction

Pancreatic cancer is a malignancy that exhibits an aggressive clinical course; e.g., the overall survival for this type of cancer is typically 6 months from diagnosis^1–4^. The 5-year relative survival rate for all stages combined remains at 8%, in contrast with the steady increase in survival observed for most other cancer types^4^. Moreover, at diagnosis, 80%–85% of patients are at stages that are unresectable and the chemotherapeutic treatments currently available for this disease are ineffective, resulting in a transition to progressive disease status in almost all cases^5^. This aggressive clinical course is the result of the invasive growth of pancreatic duct adenocarcinoma (PDAC) cells, which are defined by a histoarchitecture of tube-forming growth accompanied by the general invasion of the retroperitoneal tissue and the nerve plexus, and metastasis to lymph nodes^1^.

Studies of clinical specimens have unveiled the association between genetic alterations of the Kirsten rat sarcoma viral oncogene homolog (*KRAS*) and disease initiation, and have demonstrated the presence of clinically relevant mutations, including those in the cyclin-dependent kinase inhibitor 2A (*CDKN2A*), the tumor protein 53 (*TP53*), and the SMAD family member 4 (*SMAD4*), in association with PDAC development^1–3^. Moreover, a series of large-scale cancer genomics studies has determined the heterogeneous mutational profile of PDACs^6–9^. Conversely, a series of gene expression analyses have attempted to classify PDAC into subtypes that may allow the distinction between a favorable progression and the aggressive clinical behavior^10–13^. The classical subtype is PDAC with high expression of differentiated duct cell markers, while the remaining subtypes, including the squamous, basal-like, and quasi-mesenchymal forms, are PDAC with a diminished appearance of differentiated duct cell markers^10–13^. This robust advancement in classification based on gene expression has provided predictors of responses to therapy, such as CYP3A5; however, the nature of the signaling pathways that contribute to the clonal evolution and regulate the plasticity of tumors and the manner in which they associate and result in intratumoral heterogeneity remain unknown^6,13^.

Three-dimensional (3D) culture has been widely used to study the presence of tumor-initiating cells. It is a culture system that allows cellular self-organization into organoids and has rapidly emerged in studies of the pluripotency of pancreatic stem cells, of the carcinogenesis process, and of the stem cell niche environment during pancreatic cancer development^14–20^. Moreover, as 3D culture has the advantage of allowing drug screening and testing of therapeutic options^17^, its use may help elucidate the signaling pathways underlying the plasticity and clonal evolution observed during tumor development.

In our previous report, which described the rapid development of PDAC in an established genetically engineered mouse (GEM) model, we demonstrated that the PDAC and immortalized pancreatic ductal cell lines established from mice grew to form tubular structures and spheres in 3D culture^21^. In the present study, we used these cell lines to study the roles of TGF-β signaling in the formation of tubes in 3D culture conditions, because TGF-β signaling promoted the formation of tubular structures from spheroids consisting of mammary gland epithelial cells^22^. Moreover, an earlier study demonstrated that blocking TGF-β signaling increased the differentiation of the epithelial phenotype on the spheroids formed^16^. Here, we recapitulated the clonal growth of PDAC and immortalized pancreatic duct cell lines in 3D culture and demonstrated that tube-forming growth relies on the TGF-β-signaling-induced fading epithelial phenotype. Conversely, the elimination of TGF-β signaling diminished the tube-forming growth by increasing the number of spheres with epithelial adhesion. Furthermore, the expression of TGF-β-signaling targets was altered, with a phenotypic shift from the tube- to the sphere-forming growth in both PDAC and immortalized pancreatic duct cell lines.

## Methods

### Mice and cell lines

*TKC* and *TC* mice in the C57BL/6 background were obtained by the mating of *LBSL-tsTAg*^*Tg*/−^ mice (strain ID: T26, Institute of Medical Science of the University of Tokyo, Tokyo, Japan)^23^ with *Kras*^*LSL–G12D*/+^ and *Pdx1-Cre*^*Tg*/−^ mice (National Cancer Institute Mouse Repository, Frederick, MD, USA). *TKC* mice express both the SV40 tsA58 large T antigen (tsTAg) and Kras G12D in the pancreas and carry *LBSL-tsTAg*^*Tg*/−^, *Kras*^*LSL–G12D*/+^, and *Pdx1-Cre*^*Tg*/−^ alleles. *TC* mice have no *Kras G12D* mutation, but exhibit dysfunctions of p53 because of the expression of tsTAg. The control mouse pancreatic tissue was from C57BL/6 mice aged 10–18 weeks (CLEA Japan, Tokyo, Japan and Charles River Laboratories Japan, Yokohama, Japan). The Animal Care and Use Committee of the National Institute of Advanced Industrial Science and Technology (AIST) and Chiba University approved all animal care. The experiments were performed based on the Fundamental Guidelines for Proper Conduct of Animal Experiments and Related Activities in Academic Research Institutions under the jurisdiction of the Ministry of Education, Culture, Sports, Science and Technology of Japan.

Our previous study provided detailed information about the YamaPaca-6 and YamaPaca-25 cell lines, which are PDAC cell lines that were previously established from tumors of *TKC* mice, and about the immortalized pancreatic duct epithelial cell lines DC-11 and DC-19, which are derived from *TC* mice^21^. For maintenance, these cell lines were cultured using complete medium (high-glucose Dulbecco’s Modified Eagle’s Medium (DMEM; Wako Pure Chemical Industries, Osaka, Japan) containing 10% fetal bovine serum [FBS], 1× MITO + Serum Extender [BD Biosciences, Bedford, MA, USA], 100 U/mL of penicillin, and 100 μg/mL of streptomycin [Thermo Fisher Scientific, Waltham, MA, USA]) in type-I collagen-coated dishes (AGC TECHNO GLASS, Yoshida-Cho, Japan) at 33 °C and 5% CO_2_.

The human pancreatic cancer cell line SUIT-2 was obtained from the Cell Resource Center for Biomedical Research, Institute of Development, Aging, and Cancer, Tohoku University (Sendai, Japan). Another human pancreatic cancer cell line, Capan-1, was obtained from the American Type Culture Collection (Manassas, VA, USA). SUIT-2 cells were cultured using complete medium (low-glucose DMEM [Wako Pure Chemical Industries] containing 10% FBS, 100 U/mL of penicillin, and 100 μg/mL of streptomycin) in tissue culture dishes (TPP Techno Plastic Products AG, Trasadingen, Switzerland) at 37 °C and 5% CO_2_. Capan-1 cells were cultured using complete medium (Iscove’s modified Dulbecco’s medium [Sigma-Aldrich, St. Louis, MO, USA] containing 0.584 g/L l-glutamine [Thermo Fisher Scientific], 20% FBS, 100 U/mL of penicillin, and 100 μg/mL of streptomycin) in tissue culture dishes (TPP Techno Plastic Products AG) at 37 °C and 5% CO_2_.

### 3D culture

YamaPaca-6, YamaPaca-25, and DC-11 and DC-19 cells formed spherical and tubular structures in 3D culture using Cellmatrix Type I-A collagen (Nitta Gelatin, Osaka, Japan) and were subjected to limiting dilution assays. Cell suspensions in 10 μL of pH-neutralized Cellmatrix Type I-A collagen containing 50 cells/mL and the Cellmatrix gel were incubated in 5% CO_2_ at 37 °C for 30 min, for gelation, followed by culture using the complete medium at 33 °C and 5% CO_2_. Cells were recovered by incubation with 0.2 mg/mL (final concentration) of Collagenase L (Nitta Gelatin) at 37 °C for 10–30 min.

The formation of tubes and spheres was demonstrated in time-lapse images and movies of 3D cultures. Recombinant proteins of human/mouse/rat Activin A (R&D Systems, Minneapolis, MN, USA), mouse Nodal (R&D Systems), human BMP-2/BMP-7 (R&D Systems), human BMP-4/BMP-7 (R&D Systems), and mouse TGF-β1 (Cell Signaling Technology, Danvers, MA, USA), or the TGF-β-signaling inhibitors SB-431542 (Sigma-Aldrich) and LY-364947 (Wako Pure Chemical Industries), were added to the complete medium, and collagen samples containing cells (5 × 10^4^ cells/mL) were incubated in 5% CO_2_ at 33 °C for 8 days. Human TGF-β1 (PeproTech, Rocky Hill, NJ, USA) was used for 3D culture of human pancreatic cancer cell lines with TGF-β1 stimulation. The 3D culture of Capan-1 and SUIT-2 cells was performed at 37 °C in 5% CO_2_ for 8 days. The morphological assessment was performed on time-lapse images that were acquired using an Axio Observer Z1 microscope (Carl Zeiss, Oberkochen, Germany).

For transmission electron microscopy (TEM), 3D cultured gels were fixed for 24 h in phosphate-buffered 2.5% glutaraldehyde (Wako Pure Chemical Industries). Postfixation was performed for 1 h in 1% osmium tetroxide in 0.1 M phosphate buffer (pH 7.4) in an ice bath. The specimens were embedded in Epon 812. Ultrathin sections were obtained, stained with uranyl acetate and lead citrate, and examined using a JEM-1011 transmission electron microscope (JEOL), as described previously^24^.

### RNA purification, real-time PCR array analysis, and RNA-seq analysis

Total RNA from the 3D-cultured cells or pancreatic tissues was purified using a RNeasy Mini Kit with an RNase-free DNase Set (QIAGEN, Venlo, Netherlands), according to the supplier’s manual. The quality and amount of the RNA in each sample were evaluated using Experion (Bio-Rad, Hercules, CA, USA) and NanoDrop (Thermo Fisher Scientific), respectively.

For real-time PCR analysis, cDNAs were prepared using the RT^2^ First-Strand Kit (QIAGEN) and were subjected to real-time PCR analysis on an Applied Biosystems 7500 apparatus (Life Technologies, Carlsbad, CA, USA) using RT^2^ Profiler PCR Array plates (Mouse TGF-β-Signaling Targets #PAMM-235Z; QIAGEN) and the RT^2^ SYBR Green ROX qPCR Master Mix (QIAGEN). The QIAGEN Data Analysis Center used the *Δ*ΔCt method (threshold Ct number = 35) to obtain expression data for each gene (corrections were performed using the geometric mean of the Ct number of *Actb*, *B2m*, *Gapdh*, *Gusb*, and *Hsp90ab1*, which were used as internal standards).

For RNA-seq analysis using a next-generation sequencer (NGS), a QuantSeq 3′ mRNA Seq Library Prep Kit for Illumina (FWD) (LEXOGEN, Vienna, Austria) was used to prepare libraries. RNA sequencing was performed on a NextSeq500 (Illumina, San Diego, CA, USA) in a 75-base single-end mode. The RNA sequence data were analyzed using the Strand NGS software, v3.2 (Strand Life Sciences, Bangalore, India).

### Transplantation experiment

The transplantation experiment was performed as described previously^21^. Briefly, 1 × 10^6^ YamaPaca cells were injected into the abdominal cavity of KSN/Slc nude mice (Japan SLC, Hamamatsu, Japan) under isoflurane anesthesia (Abbott, Chicago, IL, USA). The tumors that formed were excised with a scalpel (Surgical Blade No. 11; Feather Safety Razor Co., Ltd., Osaka, Japan) and fixed in neutral-buffered 10% formalin overnight for routine processing and paraffin embedment. Each experimental group contained five mice.

### Immunohistochemistry

Analysis by immunohistochemistry was performed according to protocols described previously^25^. Briefly, 3 μm serial sections were prepared from paraffin-embedded materials and subjected to hematoxylin–eosin (H&E) staining and immunohistochemistry using antibodies, including an anti-CK-19 antibody (The Developmental Studies Hybridoma Bank, University of Iowa, Iowa City, IA, USA), an anti-TAg antibody (Santa Cruz Biotechnology, Santa Cruz, CA, USA), and an anti-Smad2/3 antibody (Cell Signaling Technology). For visualization of the immune complexes, the Vectastain Elite ABC Kit (Vector Laboratories, Burlingame, CA, USA) was used.

### Statistics

All statistical analyses were performed using BellCurve for Excel (Social Survey Research Information Co., Ltd., Tokyo, Japan).

## Results

### TGF-β signaling is more active in the formed tube structures compared with spheres

**Figure 1:**
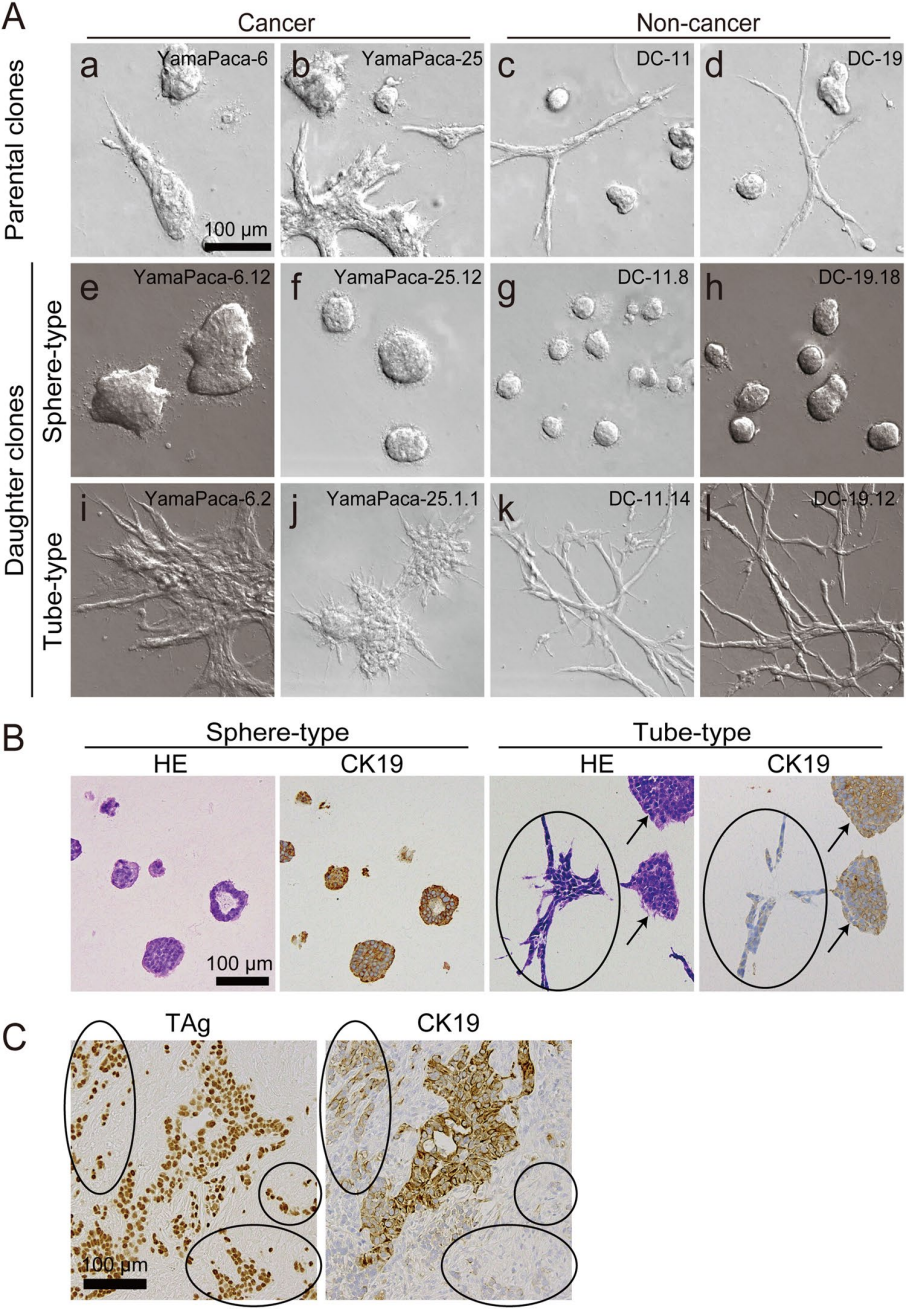
Sphere- and tube-forming clones of YamaPaca and DC cells. (**A**) Pancreatic cancer cell lines, YamaPaca-6 (a) and YamaPaca-25 (b), and immortalized pancreatic duct epithelial cell lines, DC-11 (c) and DC-19 (d), form spheres and tubes in 3D culture using type I collagen. The isolated sphere-forming cancer or immortalized cell lines are termed YamaPaca-6.12 (e) and YamaPaca-25.12 (f) or DC-11.8 (g) and DC-19.18 (h), respectively. The isolated tube-forming cancer or immortalized cell lines are termed YamaPaca-6.2 (i) and YamaPaca-25.1.1 (j) or DC-11.14 (k) and DC-19.12 (l), respectively. (**B**) Tube-forming YamaPaca cells express CK19 at a lower level compared with sphere-forming cells. The images show H&E staining and anti-CK19 immunostaining. The arrows and circles indicate the spheres and tubes, respectively, that originated from tube-forming clones. (**C**) Tube-forming YamaPaca cells express CK19 at a lower level than do sphere-forming cells in the transplanted tumor. The sphere-forming YamaPaca-6.12 clone is transplantable into the abdominal cavity of nude mice, where it develops tumor cells that express CK19. CK19 expression is downregulated in tube-forming cells (circles), which grow invasively and express TAg.

All PDAC and immortalized pancreatic duct cell lines expressed cytokeratin 19 (CK19), a pancreatic duct marker, and formed spherical and tubular structures in 3D culture using type I collagen (Fig. 1A-a–d). Both sphere- and tube-forming clones were isolated (Fig. S1) in this study, and 3D cultures showed clonal growth: sphere-forming cancer cell lines (YamaPaca-6.12 and -25.12; Fig. 1A-e,f); sphere-forming immortalized cell lines (DC-11.8 and -19.18; Fig. 1A-g,h); tube-forming cancer cell lines (YamaPaca-6.2 and -25.1.1; Fig. 1A-i,j); and tube-forming immortalized cell lines (DC-11.14 and -19.12; Fig. 1A-k,l). The results of the time-lapse imaging analysis illustrated the differential morphogenesis between spherical and tubular structures in 3D culture (Fig. S2). More precisely, tube formation was invasive, whereas sphere formation was not.

In the 3D culture, the YamaPaca-6.12 and -25.12 clones formed spherical structure, the cells of which showed higher CK19 expression than did the cells in the tube structures formed by YamaPaca-6.2 and -25.1.1 (Fig. 1B). In the transplantation experiment, YamaPaca-6.12 and -25.12 cells grew into tumors via tube formation in the abdominal cavity of nude mice, in which most areas were occupied by tube structures and exhibited marked downregulation of CK19 expression (Fig. 1C). Conversely, the area that exhibited solid and cystic growth was small and restricted and contained proliferating cells with higher CK19 expression (Fig. 1C). The tube-forming growth appeared with the downregulation of CK19 expression in sphere-forming clones, which raised the possibility that the tumor microenvironment provided an activation signaling aimed at altering the structural phenotypes, resulting in reduced CK19 expression in cells. Moreover, because earlier studies pointed out the correlation between TGF-β-signaling activation and the decreased expression of CK19 in PDAC^26^ and cholangiocarcinoma^27^, we focused on TGF-β signaling and analyzed the mRNA expression of its target genes using a real-time RT–PCR array analysis. Eleven and 25 of the total 84 target genes of TGF-β signaling were expressed at levels that were twofold higher in the tube-forming cells YamaPaca-6.2 and -25.1.1 than they were in the sphere-forming cells YamaPaca-6.12 and -25.12 (Tables 1 and S1), whereas only two and five genes exhibited a lower expression (by 0.5-fold), respectively. These results suggest the presence of a positive association between the activation of the TGF-β signaling pathway and clonal growth with a tube-forming phenotype.

**Table 1:** Comparison of TGF-β signaling target genes expressed in tube- and sphere-forming cancer cells.

Ratios of quantified mRNA expression^*a^		Number of genes		
		YamaPaca-6.2/YamaPaca-6.12	YamaPaca-25.1.1/YamaPaca-25.12	Same results between YamaPaca-6 and -25
Up	2.0<	11	25	6^*b^
Slight up	1.5–2.0	7	15	1
No change	0.75<, <1.5	55	36	NA
Slight down	0.5–0.75	8	2	1
Down	<0.5	2	5	0
Total		84	84	NA

^*a^Ratios were calculated according to the following formula: mRNA expression levels of tube-forming cells/mRNA expression levels of sphere-forming cells.

^*b^The names of the six genes are shown in Table 2.

### Activation of TGF-β signaling promotes tube formation in YamaPaca cells

**Figure 2:**
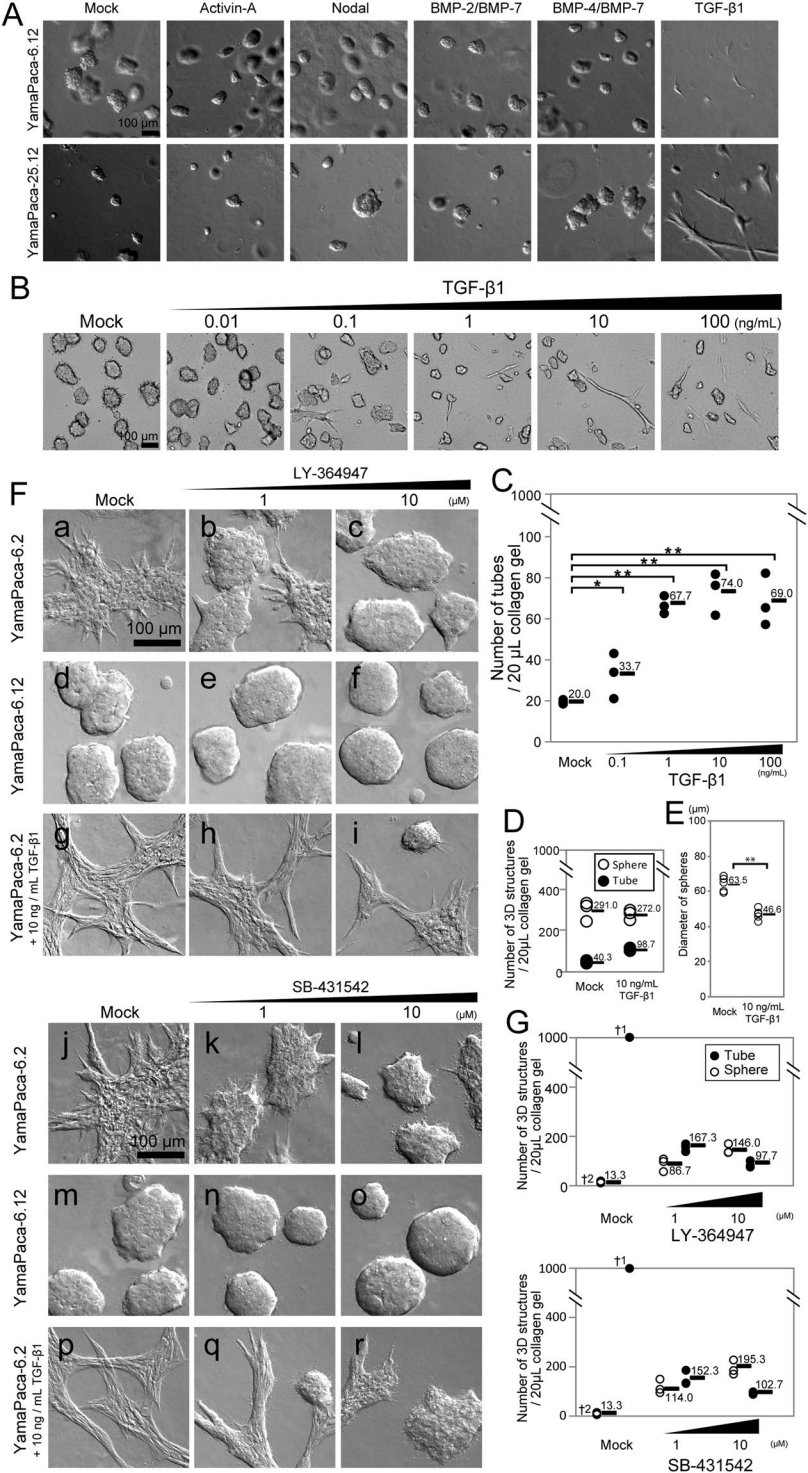
TGF-β promotes tube formation in pancreatic cancer cell lines. (**A**) TGF-β1 treatment (10 ng/mL) alters the sphere-forming capacity of the YamaPaca-6.12 clone toward tube formation. The sphere-forming YamaPaca clones were cultured in collagen for 8 days with subsequent stimulation; mock treatment was used as a negative control (100 ng/mL Activin-A, 100 ng/mL Nodal, 100 ng/mL BMP-2/BMP-7, 100 ng/mL BMP-4/BMP-7, and 10 ng/mL TGF-β1). (**B**) Dose dependency was evident for the YamaPaca-6.12 clone in the 3D culture condition. (**C**) An increasing number of tubes was observed with increasing TGF-β1 concentration in the 3D culture of YamaPaca-6.12 clones. TGF-β1 was administered to 1000 cells per 20 μL of collagen for 8 days at the following concentrations: mock, 0.1, 1, 10, and 100 ng/mL (n = 3). Multiple comparison (Williams’ method) was used for statistical analysis. *Static ≥ rejection value (5%) and ** static ≥ rejection value (2.5%) compared with the controls (mock treatment). Horizontal bars, averages. (**D**) YamaPaca-6.12 cells form tube structures by TGF-β1 stimulation. A subpopulation of cells formed tubes by TGF-β1 stimulation, while the large majority of cells did not. (**E**) The speed of the growth of sphere structures was decreased by TGF-β1 stimulation. The diameter of sphere structures under the TGF-β1 stimulation was smaller than that of the structures that received mock treatment. **P < 0.01, *t*-test. (**F**) Treatment with a TGF-β-signaling inhibitor (LY-364947 or SB-431542) alters the tube-forming YamaPaca-6.2 clone toward the formation of spheres. Increasing concentrations of the inhibitors diminish the tube-forming capacity of the YamaPaca-6.2 clone and antagonize the treatment with 10 ng/mL of TGF-β1, whereas the sphere-forming capacity of the YamaPaca-6.12 clone is not affected by treatment with TGF-β-signaling inhibitors. (**G**) The number of spheres and tubes in the 3D culture of YamaPaca-6.2 cells was counted (n = 3). ^†1^Uncountable because of tube-network generation. ^†2^Common.

Treatment of YamaPaca-6.12 and -25.12 clones with TGF-β1 yielded tube structures that coexisted with the formed spherical structures, while the administration of the other molecules of the TGF-β superfamily (activin A, Nodal, BMP-2/BMP-7 heterodimer, and BMP-4/BMP-7 heterodimer) did not affect the morphological phenotype (Fig. 2A). Increasing the concentration of TGF-β1 raised the number of formed tube structures, which never exceeded the number of coexisting spherical structures (Fig. 2B–D). Conversely, increasing the concentration of TGF-β1 decreased the average diameter of formed sphere structures compared with mock treatment (Fig. 2E).

Treatment with the TGF-β-signaling inhibitors LY-364947 or SB-431542 altered the tube-forming phenotype of YamaPaca-6.2 and -25.1.1 cells toward the sphere-forming phenotype (Fig. 2F-a–c,j–l,G). Treatment with TGF-β-signaling inhibitors dramatically diminished the tube-structure formation rate of these cells, while spheres appeared. Increasing the concentration of TGF-β-signaling inhibitors raised the number of formed spherical structures, which never exceeded 20% of the number of seeded cells (Fig. 2G). To determine whether the human PDAC cell lines exhibited similar growth phenotypes in 3D culture, we investigated Capan-1 and SUIT-2 cells. Capan-1 cells formed sphere structures and showed tube-forming growth upon TGF-β1 treatment, while SUIT-2 cells did not (Fig. S3). TGF-β1 treatment stimulated the formation of tubular structures by a part of human PDAC cell lines, which was consistent with the results of a previous study that was performed using human mammary gland epithelial cells^22^.

An antagonistic relationship between TGF-β1 and the inhibitors of TGF-β signaling was evident regarding the morphogenesis shift between the tube- and sphere-forming phenotypes (Fig. 2F-g–i,p–r). It is noteworthy that the results of the real-time PCR analysis showed the reciprocal expression of six genes (*Fn1*, *Gadd45b*, *Hey1*, *Mmp2*, *Snai1*, and *Tgfb2*; see Tables 2 and S1) among the TGF-β-signaling target genes between TGF-β1-treated sphere-forming cells (Tables 3 and S1) and the LY-364947-treated tube-forming cells (Tables 4 and S1). Detection of Tgfb2 (TGF-β2), which is a TGF-β ligand (TGF-β1–3), Mmp2, Fn1, and Snai1 is widely used as a hallmark of the activation of TGF-β1 signaling^28–30^.

**Table 2:** Genes that are highly expressed (ratio > 2.0) in tube-forming cancer cells.

Gene name	Ratio of quantified mRNA expression*	
	YamaPaca-6.2/YamaPaca-6.12	YamaPaca-25.1.1/YamaPaca-25.12
*Fn1*	2.39	4.97
*Gadd45b*	2.73	5.04
*Hey1*	3.91	42.14
*Mmp2*	6.95	21.23
*Snai1*	2.31	5.72
*Tgfb2*	3.91	17.12

*Ratios were calculated according to the following formula: mRNA expression levels of tube-forming cells/mRNA expression levels of sphere-forming cells.

**Table 3:** Changes in the expression of the six genes after treatment of sphere-forming clones with TGF-β1.

Gene name	Ratio of quantified mRNA expression*	
	YamaPaca-6.12	YamaPaca-25.12
*Fn1*	3.53	6.91
*Gadd45b*	5.58	2.94
*Hey1*	4.01	4.36
*Mmp2*	0.90	6.44
*Snai1*	10.93	8.37
*Tgfb2*	21.74	3.92

*Ratios were calculated according to the following formula: mRNA expression levels of TGF-β1-treated cells/mRNA expression levels of mock-treated cells.

**Table 4:** Changes in the expression of the six genes after treatment of tube-forming clones with LY-364947.

Gene name	Ratio of quantified mRNA expression*	
	YamaPaca-6.2	YamaPaca-25.1.1
*Fn1*	0.48	0.07
*Gadd45b*	1.01	3.19
*Hey1*	0.18	0.13
*Mmp2*	0.23	0.29
*Snai1*	0.68	0.18
*Tgfb2*	0.92	0.31

*Ratios were calculated according to the following formula: mRNA expression levels of LY-364947-treated cells/mRNA expression levels of mock-treated cells.

**Figure 3:**
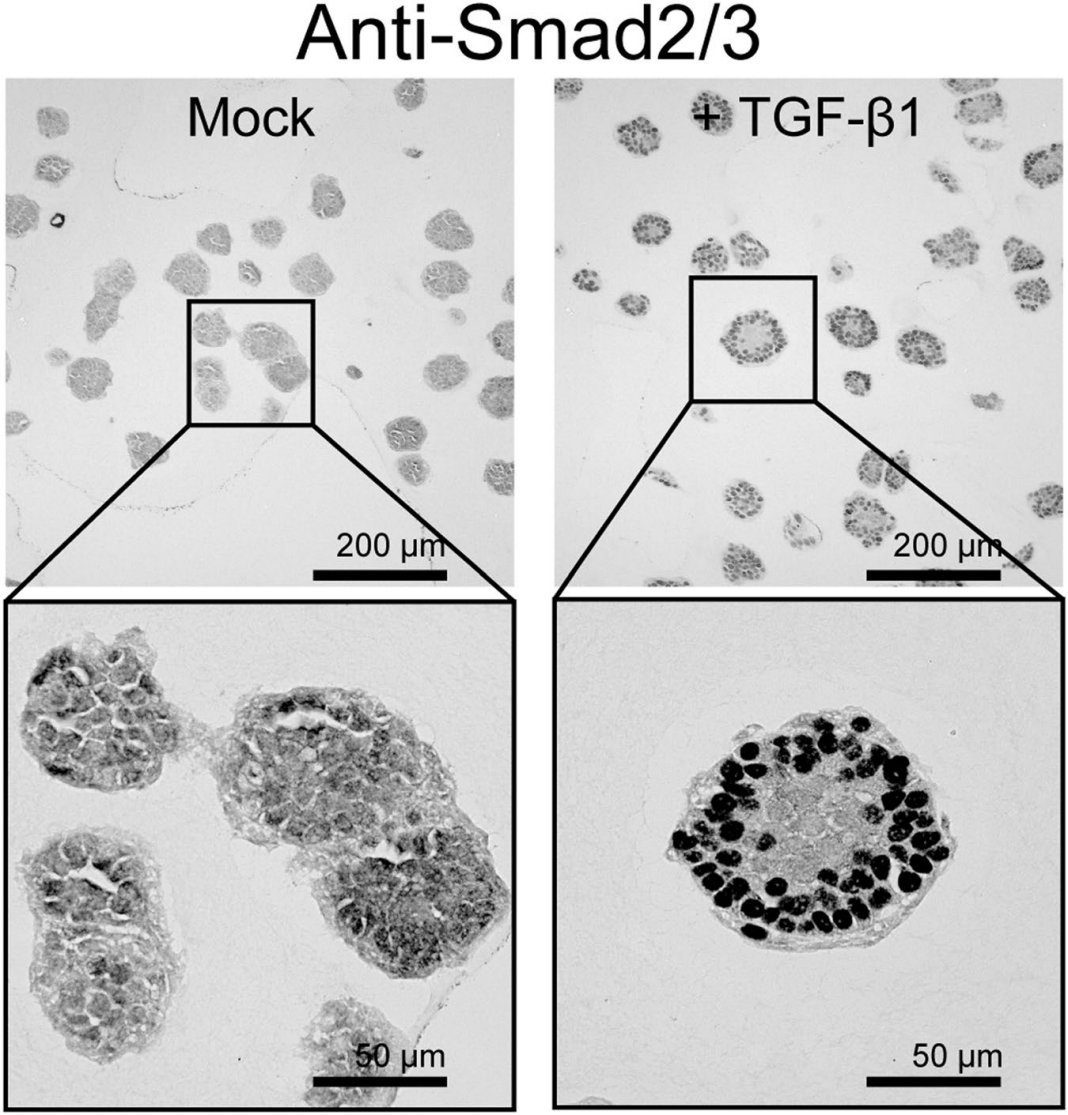
TGF-β treatment translocates Smad-2/3 into the nucleus of YamaPaca cells. Immunohistochemical detection of Smad-2/3 expression in YamaPaca-6.12 cells in 3D culture with/without stimulation using 10 ng/mL of recombinant TGF-β1 for 3 h.

Furthermore, TGF-β-signaling activation was evident in the immunohistochemical analysis. Smad-2/3 translocated from the cytoplasm to the nucleus after treatment with 10 ng/mL of recombinant TGF-β1 (Fig. 3). These results suggest that the shift in the morphogenesis of these cells from spheres to tubes is associated with a robust TGF-β-signaling activation, which is linked to the expression of its target genes. Moreover, the detection of mRNA expression may help determine whether tube-forming growth driven by TGF-β1-signaling activation is present in the pancreas.

### Tube formation decreases the epithelial features, as detected using electron microscopy

**Figure 4:**
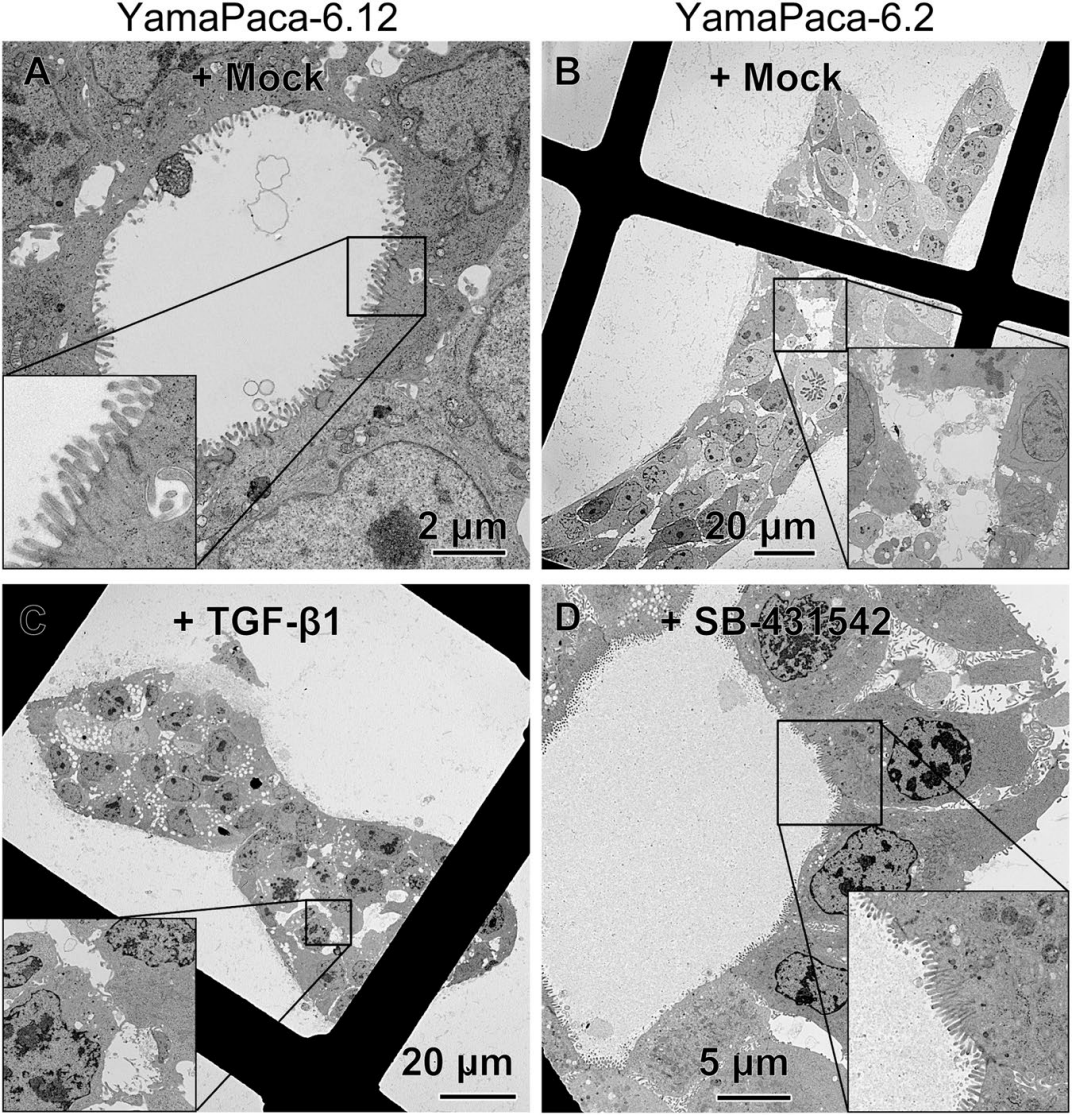
Ultrastructural appearance of tubes and spheres. Cells were cultured in collagen (5 × 10^4^ cells/mL) for 8 days and subjected to electron microscopic analysis. The images show (**A**) sphere-forming YamaPaca-6.12, (**B**) tube-forming YamaPaca-6.2, (**C**) 10 ng/mL TGF-β1-treated YamaPaca-6.12, and (**D**) 10 μM SB431542-treated YamaPaca-6.2 cells.

To identify the ultrastructural changes of YamaPaca cells in 3D culture induced by TGF-β-signaling activation, we performed an electron microscopy analysis. YamaPaca-6.12 cells formed spheres with tethered cell–cell structures and a cystic lumen coated with microvilli (Fig. 4A), whereas YamaPaca-6.2 cells formed tubular structures that displayed a loosely arranged space and random distribution of microvilli on the luminal surfaces (Fig. 4B). Figure 4A shows the findings of microvilli and tight junctions, which were typical ductal epithelial cell features in a previous study^17^. In contrast, the administration of TGF-β1 to YamaPaca-6.12 cells altered their ultrastructural appearance, with the development of a more loosely arranged space and tiny microvilli (Fig. 4C). Conversely, treatment with TGF-β-signaling inhibitors changed the ultrastructural appearance of YamaPaca-6.2 cells toward the formation of spheres that had tethered cell–cell structures and a cystic lumen coated with microvilli (Fig. 4D). These electron microscopic findings resemble the features that are observed during mammalian gastrulation^31,32^.

### Fn1 may be a fine marker *in vivo*

**Figure 5:**
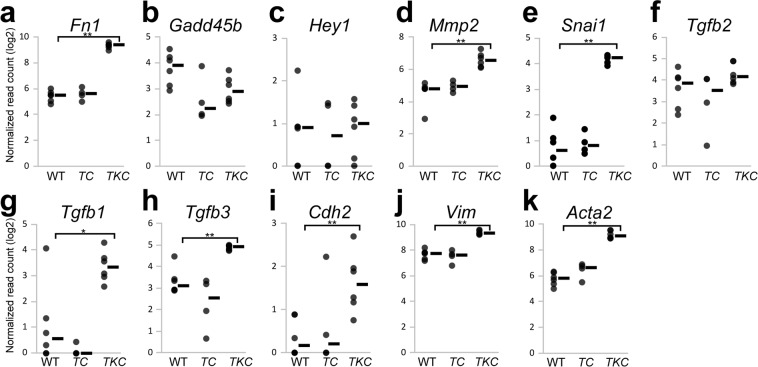
Fn1 is a fine marker *in vivo*. The expression of the six genes (**a–f**) and other genes (**g–k**) in the PDAC tissues from *TKC* mice and pancreatic tissues from *TC* and wild-type (WT) mice was analyzed by RNA-seq. Multiple comparison (Steel’s method) was used for statistical analysis. *P < 0.05, **P < 0.01, compared with WT mice. Horizontal bars, medians.

To determine whether TGF-β-signaling activation occurred with the increase of the tube-forming growth, we evaluate the mRNA expression in the pancreas of genetically engineered mice (*TKC* mice)^21^. *TKC* mice rapidly develop PDAC, the histoarchitecture of which is the massive tube-forming growth of pancreatic cancer, while wild-type and tsTAg-expressing (*TC*) mice do not develop pancreatic cancer^21^. Figure 5 shows the representative results obtained regarding the mRNA expression of the TGF-β-signaling target genes. The higher number of Fn1, MMP2, and Snai1 transcripts detected in *TKC* mice compared with WT mice suggests the existence of a correlation between the tube-forming growth of PDAC and the upregulation of transcripts including Fn1, MMP2, and Snai1 (Fig. 5a,d,e).

Given the sensitivity and reversibility of *Fn1* expression in PDAC cells in TGF-β1 *in vitro* experiments (Tables 3 and 4), the detection of Fn1 expression may help the early detection of pancreatic cancer. An analysis of the appearance of other TGF-β ligands, *Tgfb1* (Fig. 5g) and *Tgfb3* (Fig. 5h), in addition to *Tgfb2* (Fig. 5f), showed the upregulation of *Tgfb1* and *Tgfb3* in PDAC tissues of *TKC* mice. This result suggests that a TGF-β-rich environment exists in PDAC and that *Tgfb1* and *Tgfb3* play a role in the induction of invasive tube formation in PDAC tissues. Moreover, the PDAC tissues exhibited an upregulated expression of the representative mesenchymal marker genes *Cdh2* (*N-cadherin*), *Vim* (*vimentin*), and *Acta2* (*α-smooth muscle actin, α-SMA*) (Fig. 5i–k).

### The tube-forming growth of immortalized pancreatic duct cells depends on the activation of TGF-β signaling

**Figure 6:**
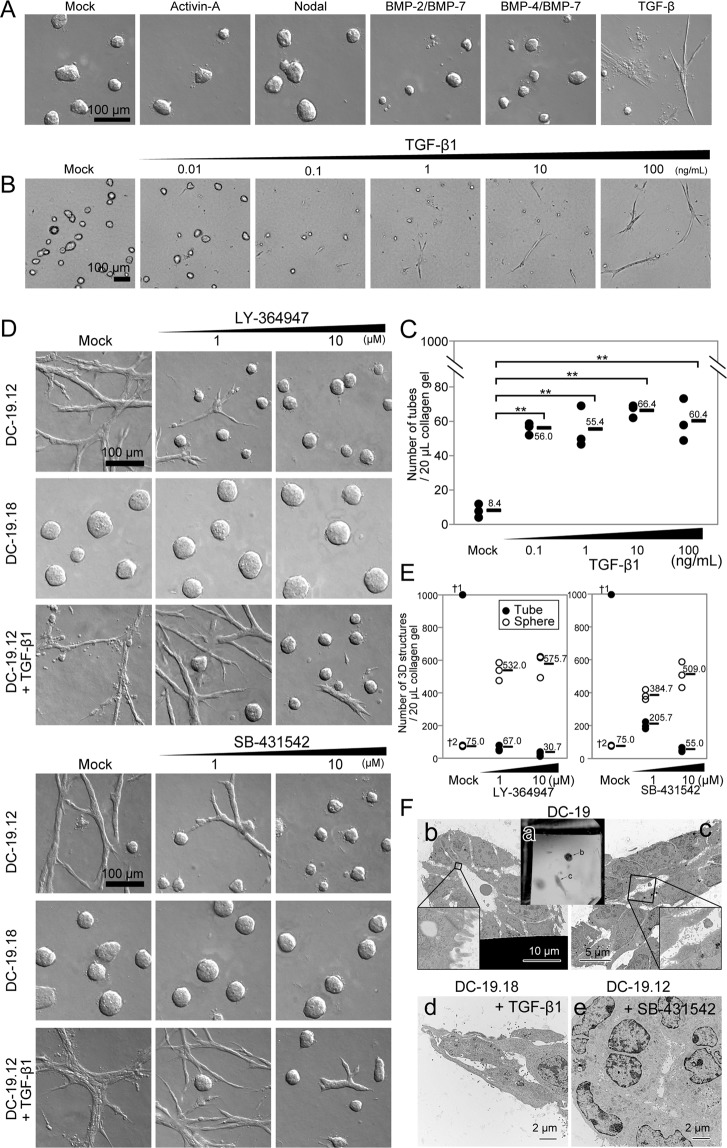
TGF-β promotes tube formation in immortalized pancreatic duct epithelial cell lines. (**A**) Treatment with 10 ng/mL of TGF-β1 alters the DC-19.18 clone from a sphere- to a tube-forming phenotype. The DC-19.18 clone was cultured in collagen for 8 days in the presence of the following molecules: negative control (mock), 100 ng/mL of Activin-A, 100 ng/mL of Nodal, 100 ng/mL of BMP-2/BMP-7, 100 ng/mL of BMP-4/BMP-7, or 10 ng/mL of TGF-β1. (**B**) Dose dependency was evident for inducing tube formation of the DC-19.18 clone in the 3D culture condition. (**C**) An increasing number of tubes was observed with increasing TGF-β1 concentration in the 3D culture of DC-19.18 cells. TGF-β1 was administered to 1000 cells per 20 μL of collagen for 8 days at the following concentrations: mock, 0.1, 1, 10, and 100 ng/mL (n = 3). Multiple comparison (Williams’ method) was used for statistical analysis. **Static ≥ rejection value (2.5%) compared with the controls (mock). Horizontal bars, averages. (**D**) Treatment with a TGF-β-signaling inhibitor (LY-364947 or SB-431542) alters the tube-forming capacity of the DC-19.12 clone toward the formation of spheres. Increasing concentrations of the inhibitors diminish the tube-forming capacity of the DC-19.12 clone and antagonize treatment with 10 ng/mL of TGF-β1, whereas the sphere-forming capacity of the DC-19.18 clone is not affected by TGF-β-signaling inhibition. (**E**) The number of spheres and tubes was counted (n = 3). Horizontal bars, means. ^†1^Uncountable because of tube-network generation. ^†2^Common. (**F**) Ultrastructural appearance of the tubes and spheres formed by immortalized pancreatic duct epithelial cell lines. The images show the macro appearance of Epon 812-embedded sphere and tube structures (a); the ultrastructural appearance of a sphere structure (b) and a tube structure (c) of DC-19 cells; a tube structure (d) of TGF-β1-treated sphere-forming DC-19.18 cells; and a sphere structure (e) of 10 μM SB-431542-treated tube-forming DC-19.12 cells.

Next, we investigated the growth of immortalized pancreatic duct cells that formed tubular structures in 3D culture. Treatment with TGF-β1, but not with the other TGF-β-superfamily molecules, altered the sphere-forming capacity of DC-19.18 cells toward the formation of tubular structures (Fig. 6A–C), whereas treatment with TGF-β-signaling inhibitors eliminated tube-forming growth in DC-19.12 clones in 3D culture (Fig. 6D,E). Furthermore, based on electron microscopic findings, the sphere structures of DC-19 cells displayed a typical ductal morphology with tethered cell–cell structures and a cystic lumen coated with microvilli (Fig. 6F-a,b)^17^. Conversely, the tubular structures of DC-19 cells displayed a loosely arranged and random distribution of microvilli on the luminal surfaces (Fig. 6F-a,c). In contrast, treatment of sphere-forming DC-19.18 cells with TGF-β1 altered their ultrastructural appearance by inducing a more loosely arranged space and tiny microvilli coating the lumen (Fig. 6F-d). Moreover, treatment with TGF-β-signaling inhibitors changed the electroscopic appearance of tube-forming DC-19.12 cells toward the formation of tethered cell–cell structures and a cystic lumen (Fig. 6F-e). These results were similar to those of the experiments that were performed using YamaPaca cells (Figs 2 and 4). It is noteworthy that immortalized cells showed reciprocal expression of *Fn1* and *Snai1* between TGF-β1-treated sphere-forming cells and the LY-364947-treated tube-forming cells (Tables S2–6). This resembles the mutual expression of *Fn1* and *Snai1* detected in YamaPaca cells. These results suggest that robust TGF-β signal activation shifts the morphogenesis of these cells from spheres to tubes in 3D culture.

## Discussion

Here, focusing on tube-forming growth, we investigated the effect of TGF-β-signaling activation using a 3D culture experiment that modeled the clonal growth of PDAC and immortalized cell lines. TGF-β signaling was more active in the tube-forming vs the sphere-forming process, while treatment of sphere-forming clones with TGF-β1 induced tube-forming growth. In fact, treatment of the sphere-forming cells with TGF-β1 activated the TGF-β signaling pathway, resulting in the nuclear accumulation of Smad-2/3, and increased the expression of TGF-β-signaling target mRNAs. It is noteworthy that TGF-β1 treatment resulted in the coexistence of tubular and spherical structures, suggesting that the differential responsiveness to TGF-β-signaling activation led to this heterogenous situation. Conversely, the elimination of TGF-β signaling via treatment with its inhibitors led to a shift from the tube-forming phenotype to the sphere-forming one and suppressed the expression of TGF-β-signaling target mRNAs. Moreover, the upregulation of the *Fn1*, *Mmp2*, and *Snai1* mRNAs, which are hallmarks of tube-forming growth in PDAC, was correlated with the increase in tube-forming growth observed in the pancreas of the mouse PDAC model. Collectively, our findings imply that TGF-β activation drives tube-forming growth and that the tube structures are a recognizable heterogeneity in 3D-cultured pancreatic cell lines.

The 3D cultures were free from stromal cells, including immune cells, fibroblasts, platelets, and the endothelial cells that form blood vessels; rather, our results highlighted the cells that could supply TGF-β to PDAC cells^33–35^. In the transplantation experiment, we showed that the sphere-forming YamaPaca-6.12 cells grew into tumors via tube formation in the abdominal cavity of nude mice, most areas of which were occupied by tube structures exhibiting downregulation of CK19 expression. These results suggest that the alteration of the growth phenotype requires signaling molecules. In this respect, TGF-β is a specific molecule in the TGF-β–cancer cell axis, and the supply of this molecule to cancer cells is correlated with an aggressive clinical course in PDAC^36^. Earlier reports pointed out that platelets release TGF-β through its activation, to promote hematogenous dissemination of PDAC^37,38^. In fact, as thromboembolic disease is a common complication of pancreatic cancer because of the generation of an intrinsic hypercoagulable state^39,40^, an earlier study suggested that the pharmacological inhibition of platelet-derived TGF-β might inhibit metastasis^37,38^. Moreover, in the carcinoma–immune cell cross-talk, the TGF-β secreted from monocytes that infiltrate the tumor tissue promotes the selective proliferation of Treg cells in the tumor stroma to suppress the antitumor immune reaction^34,41,42^. Taken together with our results, these findings suggest that the inhibition of TGF-β signaling may attenuate the rapid progression of PDAC by cutting off this cross-talk among the carcinoma, the immune cells, and platelets.

Specific therapeutic inhibition of TGF-β signaling, as a disease-rescuing agent, might result not only in the restriction of the tube-forming growth, but also in the suppression of the proliferation of PDAC cells with an epithelial–mesenchymal transition (EMT) phenotype. In fact, TGF-β is a strong inducer of EMT in PDAC cells, and pancreatic cancer cells acquire chemotherapeutic resistance through the activation of the TGF-β signaling pathway, which promotes EMT^11,43^. However, the relationship between tube-forming growth and EMT remains to be elucidated.

The difference between the results of the 3D culture and the transplantation experiment, i.e., the limited tube-forming growth driven by TGF-β signaling in 3D culture, was perplexing. In fact, the number of formed tube structures never exceeded the number of coexisting spherical-structures in 3D culture, while tube-forming growth occupied most areas of the tumor. While the detection of Fn1, MMP2, and Snai1 expression may represent a reliable method to recognize the tube-forming growth of PDAC based on the activation of TGF-β signaling, further studies are needed to understand the differential responsiveness to TGF-β-signaling activation.

Tumor classification has significance in clinical practice because it predicts the effectiveness of the chemotherapeutic options available. In this respect, our study offers the detection of the local activation of TGF-β signaling through the identification of tube-forming growth. As the clonal growth of cancer defines its response to drugs^11,12,20,44^, specific therapeutic inhibition of TGF-β signaling may be effective. In fact, there is local activation of the coagulation system and immune cell infiltration in pancreatic cancer, to a greater or lesser degree^34,37,38,41,42^, and TGF-β is provided locally, which may contribute to chemotherapeutic resistance via clonal growth in response to TGF-β-signaling activation. The identification of tube-forming growth may help support the combination of EMT inhibition with chemotherapy for PDAC treatment^45^.

We found that TGF-β treatment induced tube formation in 3D culture, which occurred over 1 week and required latent TGF-β activation. Earlier studies have shown that latent TGF-β activation occurs through Fn1 expression in osteosarcoma cells and fibroblasts^46^ and that Fn1 itself can stimulate clonal growth with a quasi-mesenchymal phenotype in mammary epithelial cells^46^. Moreover, Fn1 can mediate ES cell differentiation toward a meso-endodermal lineage^47^. In this respect, it would be interesting to define the regulatory mechanism of latent TGF-β activation. Importantly, upregulation of Fn1 occurred in the tube-forming PDAC cells, immortalized pancreatic cells, and PDAC tissues, suggesting that Fn1 expression plays an essential role in latent TGF-β activation for the maintenance of tube-forming growth. Moreover, pharmacological inhibition of Fn1 function may be effective in improving the poor prognosis of patients with PDAC because of its abundant expression and essential role in PDAC progression^48–50^. In addition, it would be interesting to understand the role of TGF-β signaling and of the upregulation of Fn1 in the tube-forming growth of immortalized pancreatic duct cell lines. Previous studies have pointed out that pancreatic epithelial cells retain their inherent plasticity substantially^17,51^, and have demonstrated the role of TGF-β signaling in acinar-to-ductal metaplasia^52^. Therefore, an analysis of the molecular function of Fn1 may help elucidate the mechanisms that balance plasticity with tube-forming growth in non-cancerous pancreatic duct cells.

In conclusion, we identified an inherent tube-forming plasticity that relied on the activation of TGF-β signaling in PDAC and immortalized pancreatic ductal cells. We highlighted the importance of the tube-forming growth in 3D culture and a mixed situation regarding the type of intratumoral heterogeneity. However, further studies are needed to assess whether the targeted inhibition of this pathway can control the aggressive clinical behavior of PDAC, and to determine the exact roles of FN1 as the target molecule of the TGF-β signaling pathway^46,53^.

## Supplementary information

Supplementary Information
