# Supplementary material for: TGF-β signaling promotes tube-structure-forming growth in pancreatic duct adenocarcinoma
Source: Sci Rep. 2019 Aug 2;9:11247. doi: 10.1038/s41598-019-47101-y (PMC6677751; doi:10.1038/s41598-019-47101-y)
Supplement: Supplementary file 1 — Supplementary Information [file 41598_2019_47101_MOESM1_ESM.pdf]

TGF-β signaling promotes tube-structure-forming growth in pancreatic duct adenocarcinoma

Takashi Yamaguchi[1, 2], Sanae Ikehara[1, 2], Yoshihiro Akimoto[3], Hayao Nakanishi[4], Masahiko Kume[5], Kazuo Yamamoto[5], Osamu Ohara[6] and Yuzuru Ikehara[1, 2]*

[1] Department of Molecular Tumor Pathology, Graduate School of Medicine, Chiba University, Chiba, 260-8670, Japan

[2] Biotechnology Research Institute for Drug Discovery, National Institute of Advanced Industrial Science and Technology (AIST), Tsukuba, 305-8565, Japan

[3] Department of Anatomy, Kyorin University School of Medicine, Mitaka, 181-8611, Japan

[4] Laboratory of Pathology and Clinical Research, Aichi Cancer Center Aichi Hospital, Okazaki, 444-0011, Japan

[5] Department of Integrated Biosciences, Graduate School of Frontier Sciences, The University of Tokyo, Kashiwa, 277-8562 Chiba, Japan

[6] Department of Applied Genomics, Kazusa DNA Research Institute, Kisarazu, 292-0818 JAPAN

*Correspondence: Yuzuru Ikehara,

Affiliation: Department of Molecular Tumor Pathology, Graduate School of Medicine, Chiba University

Address: 1-8-1 Inohana, Chuo-ku, Chiba 260-8670, Japan.

E-mail: yuzuru-ikehara@chiba-u.jp, Phone: +81-43-226-2053, FAX: +81-43-226-2058

Supplemental Text

Figure S1. Summary of the isolation of tube- or sphere-forming cells.

Three-dimensional culture was used to isolate tubes or spheres via the limiting dilution method.

Figure S2. Time-lapse images showing the growth of cancer and non-cancer cell clones in 3D culture.

The images show the growing appearance of YamaPaca-6, YamaPaca-6.2, YamaPaca-6.12, DC-19, DC-19.12, and DC-19.18 cells in 3D culture.

Figure S3. The Capan-1 cell line forms tube structures, whereas the SUIT-2 cell line does not form tube structures after TGF-β1 stimulation.

The human pancreatic cancer cell lines Capan-1(a–c) and SUIT-2 (d–f) were cultured in collagen with recombinant human TGF-β1 (10, 50 ng/mL). All cell lines formed spheres or quasi-spherical structures without TGF-β1 (a, d). Arrows: tubes induced by TGF-β1 stimulation. Culture time, 8 days.

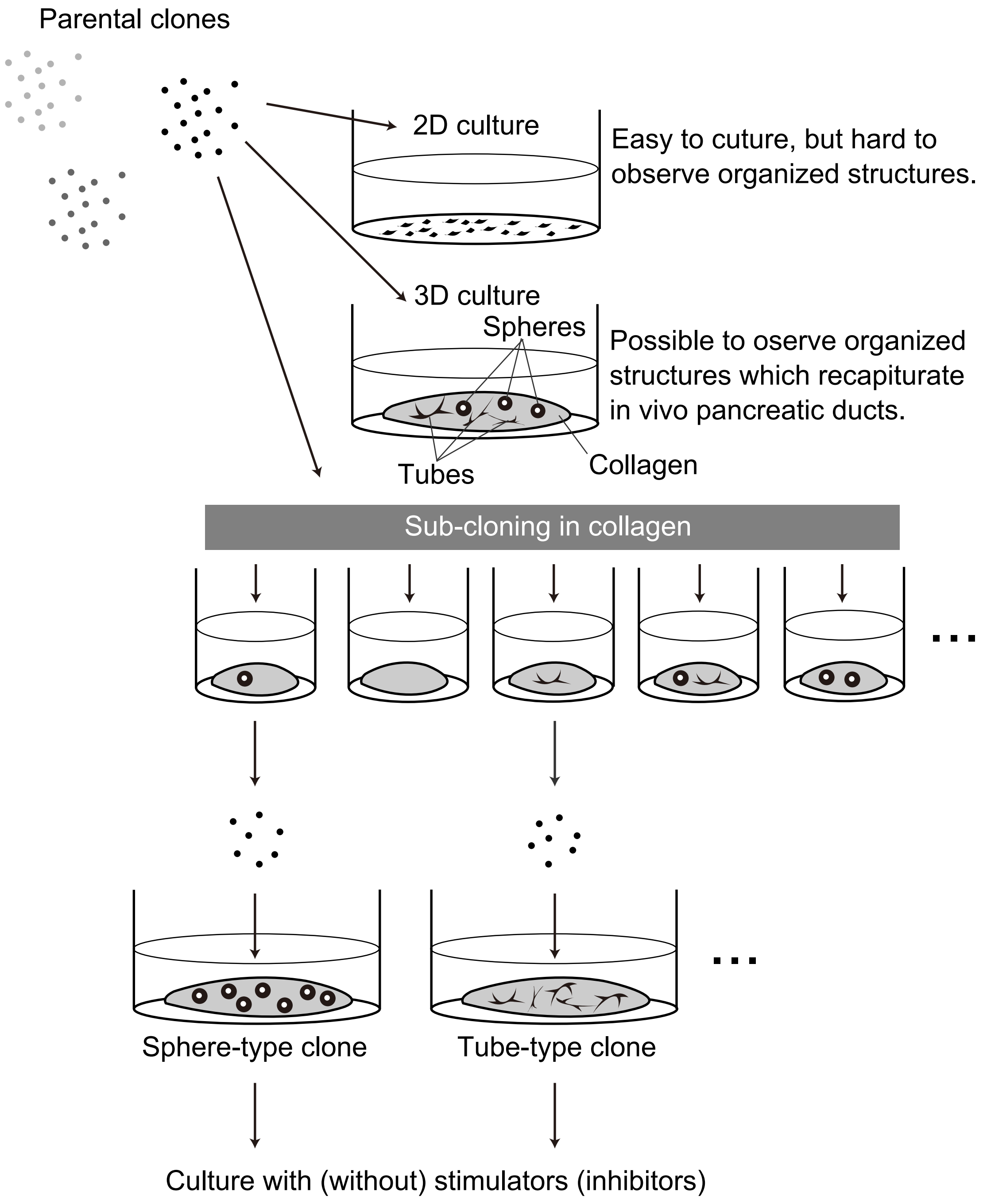

Parental clones
2D culture
Easy to cuture, but hard to
observe organized structures.
3D culture
Spheres
Possible to oserve organized
structures which recapiturate
in vivo pancreatic ducts.
Tubes
Collagen
Sub-cloning in collagen
...
...
Sphere-type clone
Tube-type clone
Culture with (without) stimulators (inhibitors)

Figure S1

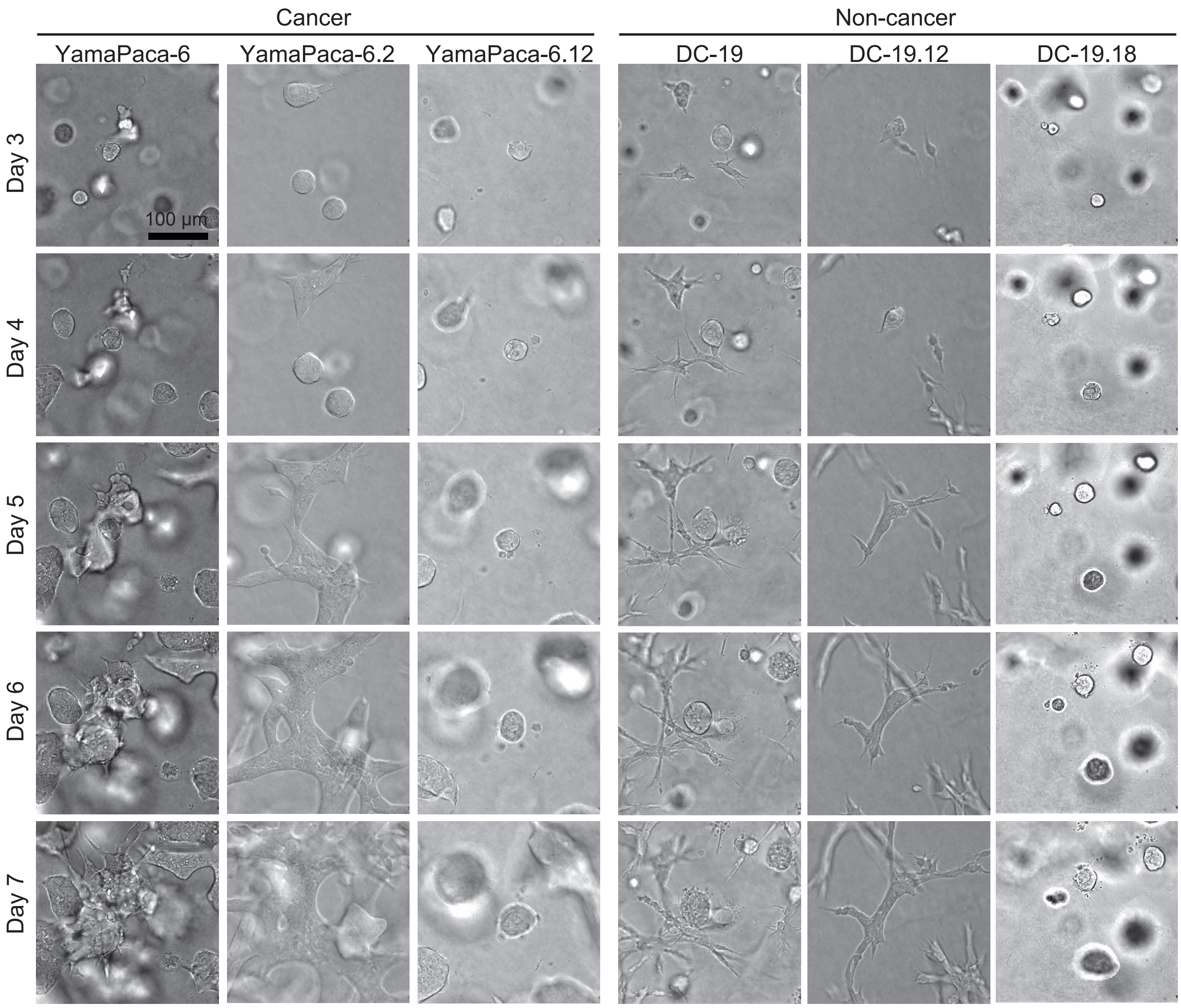
Cancer
Non-cancer
YamaPaca-6
YamaPaca-6.2
YamaPaca-6.12
DC-19
DC-19.12
DC-19.18
Day 3
Day 4
Day 5
Day 6
Day 7
100 μm

Figure S2

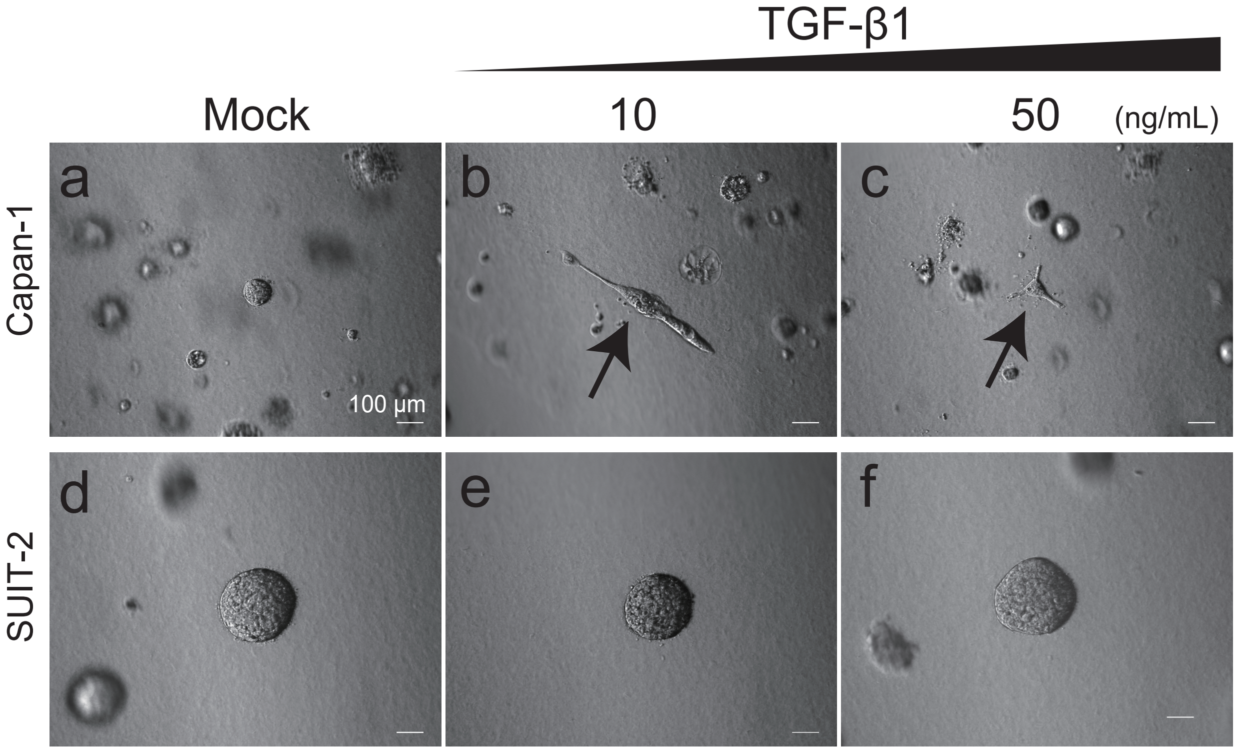

Figure S3

Table S1. Real-time PCR analyses of TGF-β signaling targets in 3D culture samples (cancer cells).

	Threshold cycle (Ct) value							
Gene name	YamaPaca-6.12	YamaPaca-6.2	YamaPaca-25.12	YamaPaca-25.1.1	YamaPaca-6.12 (TGF-β1-treated)	YamaPaca-25.12 (TGF-β1-treated)	YamaPaca-6.2 (LY-364947-treated)	YamaPaca-25.1.1 (LY-364947-treated)
Acta2	29.62	30.50	30.38	28.47	32.32	31.88	32.97	30.70
Acvr1	23.25	23.19	23.59	22.47	22.35	22.27	24.27	24.38
Acvrl1	31.26	31.16	31.70	31.37	28.66	29.17	31.84	31.54
Agt	35.00	34.27	35.00	30.66	33.96	34.36	31.29	30.91
Aipl1	27.66	27.63	28.96	28.19	27.52	28.36	28.35	29.57
Ar	25.13	25.29	24.58	28.96	25.82	26.37	26.35	32.18
Atf3	20.89	22.75	21.90	19.96	22.49	20.72	22.92	20.26
Atf4	20.88	20.54	22.13	21.40	21.32	21.60	21.16	22.17
Bach1	30.56	30.36	31.17	29.91	29.80	29.80	31.83	31.94
Bcl2l1	22.42	22.61	21.94	22.28	23.01	22.41	22.84	23.44
Bdnf	23.11	23.00	23.64	23.24	26.88	24.81	25.26	24.60
Bhlhe40	22.82	22.68	23.01	22.82	21.53	21.76	23.87	22.64
Brd2	21.19	21.33	22.32	21.63	21.20	21.78	22.46	23.54
Cdc6	23.80	23.87	24.56	24.53	24.01	24.48	25.18	26.46
Cdkn1b	20.68	21.32	22.87	21.86	22.23	22.84	22.37	22.26
Cebpb	24.25	24.20	24.74	22.32	23.46	23.95	25.15	23.40
Creb1	23.44	23.69	23.89	23.66	24.27	24.36	25.25	25.73
Crebbp	23.16	23.19	23.69	22.85	23.76	23.64	24.12	26.59
Cryab	25.89	25.92	25.21	24.47	23.54	23.64	25.50	25.31
Ctnnb1	19.12	19.43	19.84	19.36	19.22	19.76	19.90	20.56
Dnaja1	20.45	20.48	20.68	20.28	20.62	20.66	22.02	21.93
E2f4	22.67	22.84	23.28	23.32	22.95	23.28	23.15	24.62
Emp1	18.60	19.41	19.15	19.16	19.32	19.96	19.97	19.80
Eng	33.65	33.21	34.23	33.45	30.43	31.68	33.77	35.00
Ep300	23.01	23.31	24.43	23.58	24.50	24.92	24.77	27.55
Ephb2	33.57	32.89	25.26	32.70	25.72	23.55	33.44	35.00
Fn1	21.77	20.70	21.71	19.34	19.79	18.48	22.24	24.01
Fos	20.91	23.15	21.73	19.95	23.26	20.76	22.43	20.81
Furin	23.78	22.80	22.47	22.26	22.02	21.40	23.93	24.30
Gadd45b	25.59	24.33	26.26	23.86	22.95	24.26	24.79	22.93
Gli2	35.00	35.00	35.00	35.00	35.00	35.00	35.00	35.00
Gtf2i	22.28	22.72	22.52	22.35	21.94	21.86	22.76	24.65
Herpud1	25.72	24.96	28.96	27.29	27.75	28.14	26.18	26.54
Hes1	24.42	23.31	23.02	22.32	21.54	22.45	24.28	23.43
Hey1	30.65	28.87	34.41	28.94	28.49	31.84	31.85	32.59
Hmox1	24.92	24.74	25.82	25.16	23.78	23.31	25.68	25.30
Id1	20.02	20.93	21.36	20.87	23.43	22.84	22.11	21.55
Id2	20.74	20.82	21.63	21.18	21.50	24.39	22.37	23.42
Id3	23.70	23.90	25.88	23.26	24.77	25.35	24.66	23.74
Ifrd1	20.59	20.90	22.16	21.78	21.51	21.81	22.40	21.52
Il10	35.00	34.35	35.00	34.42	35.00	34.89	35.00	35.00
Klf10	23.49	23.49	25.30	23.23	23.47	24.75	24.82	23.50
Map3k7	22.34	22.20	22.56	22.70	22.67	22.75	23.61	24.37
Mapk14	22.51	23.24	23.98	23.52	23.00	24.15	23.91	24.89
Mapk8	23.18	23.36	23.41	22.98	23.94	23.91	25.17	24.51
Mbd1	23.64	24.24	24.71	24.25	23.69	24.72	24.68	25.95
Mmp2	35.00	32.39	34.78	30.31	35.00	31.65	35.00	32.85
Msx2	35.00	35.00	30.13	28.81	35.00	26.94	35.00	33.69
Myc	22.18	22.42	22.82	22.14	21.99	22.53	22.71	23.72
Myod1	35.00	35.00	35.00	35.00	35.00	35.00	35.00	35.00
Nfib	22.15	21.86	22.37	22.26	23.35	22.73	23.27	25.47
Nfkbia	18.96	18.97	20.76	18.66	19.52	20.42	20.19	18.33
Notch1	24.00	24.15	24.78	23.49	24.92	25.37	24.50	27.15
Pdgfa	30.41	27.87	24.26	25.18	25.41	22.56	28.78	26.25
Plg	35.00	35.00	34.87	35.00	35.00	35.00	35.00	35.00
Ppara	28.53	29.41	34.15	32.32	29.17	33.25	30.47	32.89
Ptgs2	20.92	21.11	21.85	21.94	21.11	20.59	22.55	21.99
Pthlh	28.21	27.66	26.76	26.37	25.78	24.39	27.43	25.69
Ptk2	22.25	22.37	22.95	22.91	22.31	22.98	22.92	24.57
Ptk2b	24.88	24.95	24.25	23.43	23.65	23.57	25.40	25.82
Rad21	20.54	20.87	21.86	21.31	21.24	22.40	22.21	22.97
Rara	23.99	24.42	24.37	23.63	23.88	23.87	24.83	25.63
Rbl1	22.00	22.18	22.90	22.48	22.60	23.18	23.14	23.40
Rhoa	18.81	19.18	19.58	19.18	18.75	19.51	20.20	19.98
Rhob	21.76	21.83	22.33	20.19	19.80	20.23	21.85	20.85
Runx1	22.95	22.84	22.71	23.01	22.20	21.44	23.75	25.67
S100a8	32.59	32.33	29.96	31.66	30.25	30.79	33.12	29.41
Serpine1	20.64	21.15	22.20	21.43	21.01	19.89	22.92	20.71
Shh	34.13	32.30	35.00	34.37	35.00	35.00	33.93	35.00
Smad1	23.92	23.89	24.55	23.41	23.91	23.79	24.94	25.78
Smad3	22.81	23.02	22.98	22.41	22.45	22.88	23.67	25.26
Smad5	23.80	23.65	24.20	23.43	23.56	24.21	24.90	25.75
Smad6	26.39	26.20	29.61	25.77	29.12	29.24	27.99	28.15
Snai1	35.00	33.98	34.29	31.71	31.39	30.79	35.00	34.96
Sox4	24.44	23.94	24.46	22.84	23.07	23.12	25.00	26.16
Sp1	22.58	22.77	23.52	23.22	23.55	24.02	23.94	25.53
Srebf2	20.62	21.18	21.72	21.17	20.71	21.73	21.43	22.79
Tgfb2	31.55	29.78	31.14	26.97	26.96	28.72	30.36	29.42
Tgfbr2	22.72	23.30	22.50	22.76	23.73	23.29	23.38	23.69
Thbs1	26.49	24.93	24.21	25.38	23.46	22.41	25.86	28.27
Tnfsf10	35.00	35.00	35.00	33.23	35.00	35.00	35.00	34.36
Txnip	22.31	23.16	25.00	24.81	25.36	24.66	24.19	22.87
Vegfa	21.58	22.38	22.62	22.99	21.91	21.01	24.18	23.55
Wfs1	24.36	24.57	21.54	25.24	23.20	23.40	24.70	27.21
Actb *	15.89	16.35	16.10	15.93	15.81	15.91	16.56	17.25
B2m *	21.21	20.91	21.51	20.99	19.70	19.37	21.13	21.57
Gapdh *	16.00	16.14	18.20	18.22	17.37	18.49	17.30	17.96
Gusb *	23.35	23.73	22.83	23.12	23.38	22.82	24.30	24.49
Hsp90ab1 *	18.41	18.61	18.69	18.78	17.58	18.41	18.73	19.56

Threshold cycle (Ct) values of all real-time PCR data obtained in this study from 3D culture samples (for Tables 1–4). *: Actb, B2m, Gapdh, Gusb and Hsp90ab1 are internal control genes.

Table S2. Real-time PCR analyses of TGF-β signaling targets in 3D culture samples (non-cancer cells)

Gene name	Threshold cycle (Ct) value							
	DC-11.8	DC-11.14	DC-19.18	DC-19.12	DC-11.8 (TGF-β1-treated)	DC-19.18 (TGF-β1-treated)	DC-11.14 (LY-364947-treated)	DC-19.12 (LY-364947-treated)
Acta2	30.42	26.73	29.31	24.12	26.93	26.85	25.90	24.49
Acvr1	24.00	23.43	23.50	23.32	21.30	20.87	23.33	23.90
Acvrl1	31.00	31.84	32.31	31.09	30.61	31.12	31.10	29.35
Agt	31.34	30.93	27.70	30.94	31.17	34.38	27.38	27.81
Aipl1	28.55	27.62	28.60	27.90	28.20	28.85	26.77	27.36
Ar	35.00	35.00	35.00	27.34	34.90	35.00	30.92	28.37
Atf3	19.79	21.56	20.44	22.28	19.76	20.16	19.87	20.18
Atf4	20.97	20.88	20.66	20.68	19.77	19.75	20.19	20.52
Bach1	29.76	30.10	29.34	29.77	28.57	28.81	28.17	29.49
Bcl2l1	22.98	22.81	23.26	22.99	23.18	23.19	22.49	22.50
Bdnf	23.71	24.32	25.12	23.15	24.22	24.84	23.69	24.34
Bhlhe40	21.81	23.00	21.91	22.85	20.83	21.13	22.01	22.92
Brd2	22.39	21.59	21.52	20.97	20.37	19.94	20.56	21.49
Cdc6	24.86	24.32	24.82	23.72	23.39	23.47	23.60	24.60
Cdkn1b	22.02	22.30	22.50	21.92	21.83	22.03	21.34	21.82
Cebpb	21.77	21.24	20.47	21.63	21.77	21.48	20.62	20.63
Creb1	25.21	24.43	24.85	23.89	23.39	23.49	23.92	24.91
Crebbp	23.97	23.83	23.89	23.60	22.47	22.51	23.26	22.99
Cryab	22.38	21.16	22.60	22.20	22.00	21.46	21.01	20.94
Ctnnb1	18.87	19.44	18.74	19.99	18.53	18.66	19.49	20.33
Dnaja1	20.63	20.44	20.46	19.72	19.58	19.65	19.97	20.42
E2f4	23.48	23.21	23.61	22.98	22.40	22.34	22.85	23.00
Emp1	19.86	19.34	20.78	18.91	19.01	18.95	20.19	20.34
Eng	34.75	35.00	35.00	32.72	32.37	30.23	32.64	33.72
Ep300	24.57	24.13	24.67	23.53	22.80	22.81	22.88	23.84
Ephb2	34.14	31.85	34.88	28.40	26.26	28.57	33.39	30.95
Fn1	23.36	20.15	21.77	18.99	17.54	17.76	20.15	20.17
Fos	20.34	22.66	21.46	23.52	19.97	20.26	20.46	19.59
Furin	23.27	23.30	23.15	23.87	21.32	21.15	23.15	23.69
Gadd45b	21.00	21.35	21.17	21.57	21.33	21.40	19.39	19.84
Gli2	35.00	29.29	35.00	27.64	35.00	35.00	27.57	27.50
Gtf2i	23.37	22.90	23.23	22.95	21.81	21.83	22.24	22.86
Herpud1	23.81	25.54	24.50	26.69	25.75	25.80	24.58	25.53
Hes1	22.66	23.80	23.31	23.54	21.77	21.82	22.82	22.83
Hey1	28.65	26.96	28.90	28.24	27.55	28.26	28.76	29.94
Hmox1	23.80	22.87	21.99	24.11	22.29	21.27	22.61	23.24
Id1	21.16	21.65	21.63	21.93	22.19	22.18	20.33	20.91
Id2	20.68	21.85	21.80	20.75	21.39	21.99	21.25	21.57
Id3	23.86	23.23	23.64	23.31	23.85	24.28	21.90	21.86
Ifrd1	21.36	22.39	21.52	21.26	20.79	21.22	21.88	21.94
Il10	35.00	35.00	35.00	34.60	33.48	33.37	33.22	33.61
Klf10	22.81	22.79	23.40	22.99	22.19	22.53	21.87	23.01
Map3k7	23.28	22.55	23.25	21.94	21.79	21.88	22.19	22.65
Mapk14	23.74	23.45	23.14	23.24	23.00	22.75	22.74	23.48
Mapk8	24.47	24.81	24.86	24.15	23.53	23.74	24.15	25.21
Mbd1	23.99	23.97	23.88	24.28	23.01	23.18	23.13	23.95
Mmp2	35.00	35.00	34.78	35.00	35.00	35.00	35.00	35.00
Msx2	29.30	31.79	29.92	33.57	27.19	27.40	33.30	32.36
Myc	20.86	21.67	20.90	22.47	20.41	20.82	20.33	21.21
Myod1	35.00	35.00	35.00	35.00	31.71	35.00	34.99	35.00
Nfib	23.29	22.15	23.24	22.17	21.65	21.90	21.62	22.16
Nfkbia	17.89	18.22	17.98	18.00	18.14	18.55	17.34	17.61
Notch1	24.78	23.91	23.88	24.58	23.56	23.26	22.90	23.19
Pdgfa	24.83	24.82	25.40	25.01	23.40	23.64	25.01	24.80
Plg	35.00	33.78	35.00	33.44	34.24	34.85	35.00	35.00
Ppara	27.57	29.84	28.01	30.66	28.34	29.20	28.23	29.35
Ptgs2	21.59	21.53	20.83	20.87	19.94	19.66	20.56	20.91
Pthlh	33.16	26.76	31.70	27.45	27.42	28.16	24.36	25.18
Ptk2	23.32	22.49	23.02	22.45	22.24	22.17	22.60	22.96
Ptk2b	24.59	25.41	24.68	25.44	23.17	23.01	25.42	25.25
Rad21	21.82	21.41	22.21	20.64	20.82	21.16	20.83	21.83
Rara	23.95	23.74	24.02	23.59	22.71	22.66	23.44	23.49
Rbl1	22.86	22.36	22.64	21.93	22.13	21.91	21.90	22.63
Rhoa	20.22	19.72	20.31	18.82	18.82	19.02	19.68	35.00
Rhob	19.98	21.18	20.21	22.86	19.44	20.12	21.02	22.28
Runx1	24.26	24.20	25.31	23.46	20.98	21.29	23.70	23.48
S100a8	34.39	35.00	34.60	34.40	32.88	35.00	33.67	34.67
Serpine1	21.60	19.69	19.79	21.43	19.33	18.83	21.26	22.30
Shh	33.54	35.00	35.00	35.00	34.99	35.00	34.39	35.00
Smad1	23.81	23.89	24.31	23.81	22.63	23.25	23.57	24.46
Smad3	23.62	23.17	22.79	23.44	22.43	22.64	23.03	23.36
Smad5	25.37	24.11	24.59	23.60	23.61	23.03	23.62	24.41
Smad6	25.57	25.77	25.79	25.54	25.65	25.74	24.94	25.46
Snai1	35.00	31.50	33.98	30.10	27.53	26.41	31.91	30.67
Sox4	24.17	23.66	23.48	24.94	22.42	22.40	23.89	24.79
Sp1	23.63	23.25	23.87	22.70	22.57	22.71	22.63	22.94
Srebf2	20.77	21.17	20.86	21.99	20.26	20.52	21.15	21.51
Tgfb2	24.48	25.47	24.52	26.33	24.42	24.77	24.63	24.71
Tgfbr2	22.74	22.27	22.87	23.20	22.80	23.18	21.96	22.00
Thbs1	25.16	21.84	23.62	21.50	20.78	20.61	20.45	19.99
Tnfsf10	34.21	35.00	35.00	35.00	35.00	35.00	32.74	32.49
Txnip	23.28	24.35	24.26	25.51	23.71	23.67	22.71	22.85
Vegfa	22.87	23.62	22.50	22.33	19.99	19.71	25.08	23.55
Wfs1	25.55	25.76	26.18	25.72	24.65	24.65	25.41	25.53
Actb *	16.54	16.31	16.53	16.31	15.41	15.52	16.62	16.91
B2m *	20.31	19.38	19.24	19.82	19.56	19.25	18.45	18.54
Gapdh *	18.27	17.85	18.41	17.77	17.95	17.90	18.17	18.40
Gusb *	23.49	23.34	23.84	22.94	22.49	22.83	22.64	25.14
Hsp90ab1 *	19.13	18.24	18.33	18.49	17.60	17.55	18.21	18.74

Threshold cycle (Ct) values of real-time PCR data obtained in this study from 3D culture samples (for Tables S3–6). *: Actb, B2m, Gapdh, Gusb and Hsp90ab1 are internal control genes.

Table S3. Comparison of TGF-β signaling target genes expressed in tube and sphere forming non-cancer cells.

Ratios of quantified mRNA expression[*1]		Number of genes: DC-11.14 / DC-11.8	DC-19.12 / DC-19.18	Same results between DC-11 and -19
Up	2.0 <	9	17	7[*2]
Slight up	1.5 – 2.0	4	9	2
No change	0.75 <, < 1.5	23	30	NA
Slight down	0.5 – 0.75	25	11	4
Down	< 0.5	23	17	11
Total		84	84	NA

*1 Ratios were calculated according to the following formula: mRNA expression levels of tube-forming cells / mRNA expression levels of sphere-forming cells.
*2 The names of the seven genes are shown in Table S4.

Table S4. Genes that are highly expressed (ratio > 2.0) in tube-forming non-cancer cells.

	Ratio of quantified mRNA expression*	
Gene name	DC-11.14 / DC-11.8	DC-19.12 / DC-19.18
Acta2	8.92	31.99
Ephb2	3.41	78.95
Fn1	6.44	6.06
Gli2	36.36	145.04
Pthlh	58.76	16.82
Snai1	7.84	12.93
Thbs1	6.89	3.83

* Ratios were calculated according to the following formula: mRNA expression levels of tube-forming cells / mRNA expression levels of sphere-forming cells.

Table S5. Changes in the expression of the seven genes after treatment of sphere-forming clones with TGF-β1.

Gene name	Ratio of quantified mRNA expression*	
	DC-11.8	DC-19.18
Acta2	5.78	3.45
Ephb2	122.01	50.23
Fn1	29.18	10.17
Gli2	0.52	0.63
Pthlh	27.68	7.35
Snai1	91.38	120.03
Thbs1	10.70	5.07

* Ratios were calculated according to the following formula: mRNA expression levels of TGF-β1-treated cells / mRNA expression levels of mock-treated cells.

Table S6. Changes in the expression of the seven genes after treatment of tube-forming clones with LY-364947.

Gene name	Ratio of quantified mRNA expression*	
	DC-11.14	DC-19.12
Acta2	1.59	1.04
Ephb2	0.30	0.23
Fn1	0.89	0.59
Gli2	2.93	1.46
Pthlh	4.69	6.45
Snai1	0.67	0.90
Thbs1	2.34	3.80

* Ratios were calculated according to the following formula: mRNA expression levels of LY-364947-treated cells / mRNA expression levels of mock-treated cells.
